# P-1866. Clinically Significant Adverse Events Rates for Daptomycin Outpatient Parenteral Antimicrobial Therapy (OPAT)

**DOI:** 10.1093/ofid/ofaf695.2035

**Published:** 2026-01-11

**Authors:** Jordan Kuck, Richard Hankins, Shawnalyn Sunagawa, Sandra A Frimpong, Nicolas W Cortes-Penfield, Bryan T Alexander, Elizabeth Lyden, Melissa LeMaster, Molly M Miller

**Affiliations:** Nebraska Medical Center, Omaha, Nebraska; Nebraska Medicine, Omaha, Nebraska; University of Nebraska Medical Center, Omaha, NE; University of Nebraska Medical Center, Omaha, NE; University of Nebraska Medical Center, Omaha, NE; Nebraska Medicine, Omaha, Nebraska; University of Nebraska Medical Center, Omaha, NE; Nebraska Medicine, Omaha, Nebraska; Nebraska Medicine, Omaha, Nebraska

## Abstract

**Background:**

Daptomycin is commonly used for outpatient parenteral antimicrobial therapy (OPAT); however, there is a gap in literature quantifying the utility of routine laboratory monitoring in this setting. Our aim was to assess adverse event (AE) rates for daptomycin to better define appropriate OPAT laboratory monitoring.

**Figure:**
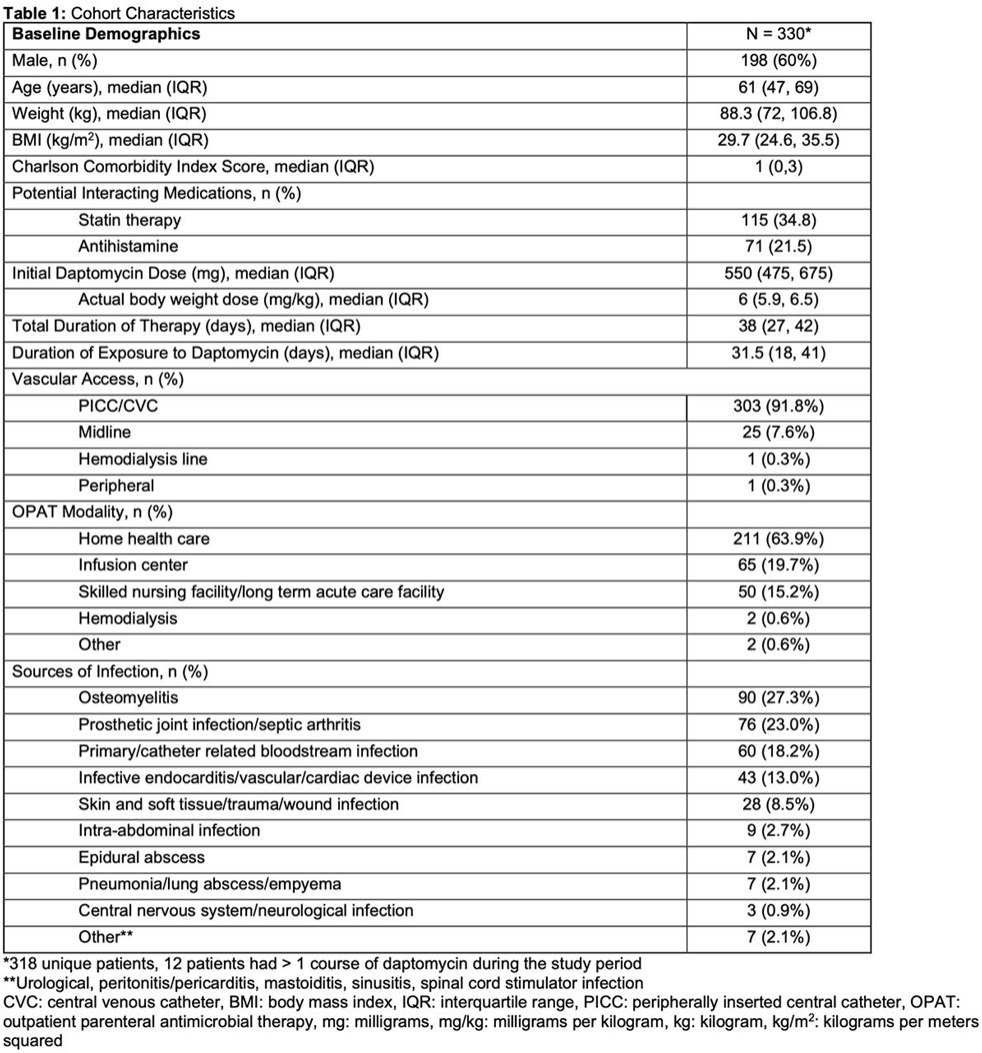

**Figure:**
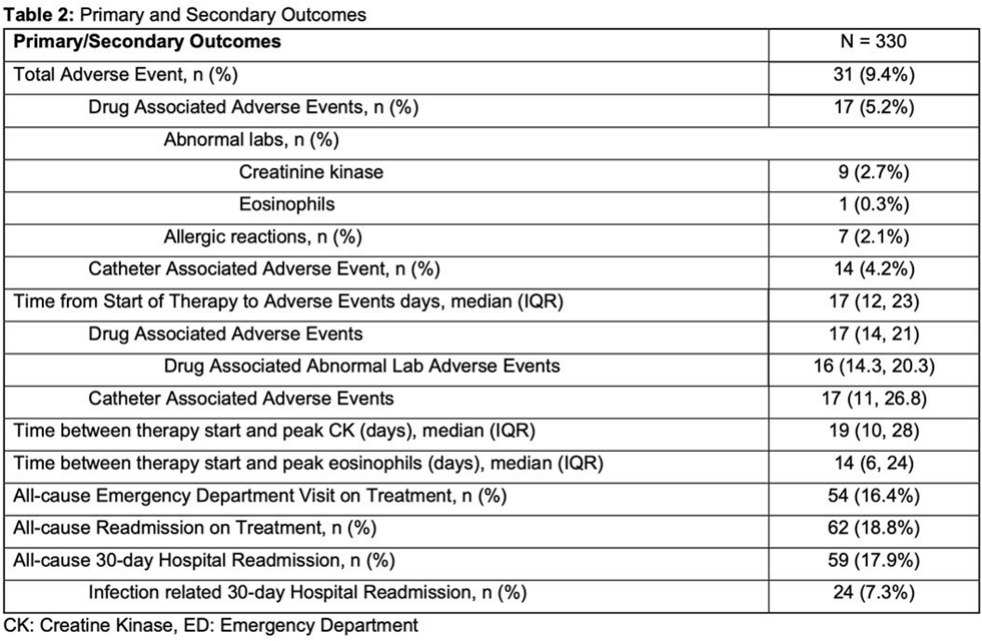

**Methods:**

We retrospectively evaluated patients who received daptomycin for a minimum of 3 days via OPAT from 6/1/2020-6/30/2024. Combination intravenous antimicrobial therapies were excluded. The primary outcome was incidence of clinically significant OPAT-related AEs, defined as drug-associated AE leading to treatment alterations (e.g. abnormal labs [drug-associated lab-related AE], allergic reactions, *Clostridioides difficile* infection) or any catheter-associated AE. Secondary outcomes included time from start of therapy to clinically significant AEs, unplanned healthcare utilization (e.g. emergency department visits, readmissions), and factors associated with increased risk of clinically significant AEs. We performed descriptive statistics for cohort characteristics, Fisher’s exact and independent sample t-test for associations with primary and secondary outcomes, and univariate and multivariate regressions for predictors of AEs.

**Figure:**
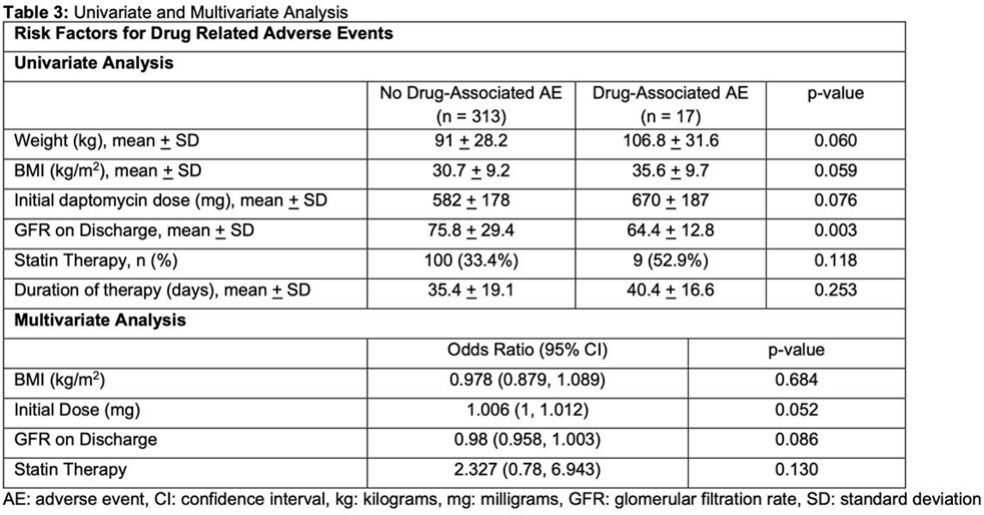

**Results:**

Cohort characteristics are summarized in Table 1. Primary and secondary outcomes are presented in Table 2. Across 330 courses of therapy, a total of 1526 sets of weekly laboratory tests were obtained, 97.2% of which were reviewed within 72 hours. Laboratory monitoring identified 10 drug-associated lab-related AEs, corresponding to 1 event per 153 lab draws. Median time from initiation of therapy to clinically significant drug-associated AE was 17 days (IQR 14, 21). Univariate and multivariate regressions are listed in Table 3. In the multivariate analysis, there were no statistically significant predictors of drug-associated AEs.

**Conclusion:**

Daptomycin was well tolerated in the OPAT setting with few drug-associated AEs, mainly occurring beyond 2 weeks of therapy. Routine weekly laboratory monitoring for daptomycin in OPAT may be more frequent than necessary, particularly in short courses.

**Disclosures:**

Bryan T. Alexander, PharmD, BCIDP, AAHIVP, Astellas Pharma: Advisor/Consultant|Merck: Grant/Research Support

